# Body stuffing during apprehension resulting in distal esophageal impaction: a case report and review of the literature

**DOI:** 10.1186/s13256-022-03628-9

**Published:** 2022-11-04

**Authors:** Tegan Schmidt, Yuliya Matolina, Arianna S. Neeki, Caros Peace, Benjamin Archambeau, Fanglong Dong, Michael M. Neeki

**Affiliations:** 1 Department of Emergency Medicine, Arrowhead Regional Medical Center, 400 N. Pepper Ave, Colton, CA 92324 USA; 2 Department of Probations, San Bernardino County, San Bernardino, CA 92415 USA; 3 California University of Science and Medicine, Colton, CA 92324 USA

**Keywords:** Esophageal foreign body, Body stuffing, Pericarditis, Abdominal pain, Chest pain

## Abstract

**Background:**

Body stuffing and body packing are two methods of concealing illicit drugs. Body stuffing is defined as the oral ingestion of illicit drugs, typically to avoid law enforcement detection or other consequences of possession, and may present a serious medical emergency in patients. Most commonly, body stuffers ingest possibly large or unknown quantities of illicit substances to avoid detection of the drugs during apprehension. This ingestion is typically hasty or impulsive, and therefore the substances ingested are rarely packaged in a way that would be considered safe for ingestion.

**Case presentation:**

This case highlights a series of rare complications of impacted esophageal foreign body including esophageal edema, pericarditis, and hydro-pneumothorax for a 16-year-old Hispanic male who was booked into a county juvenile detention and rehabilitation facility. He complained of persistent intractable epigastric pain, along with pleuritic chest pain with multiple episodes of vomiting over the previous 4 days. He denied swallowing any foreign body. He underwent esophagogastroduodenoscopy, and a plastic bag with content suspicious for marijuana was discovered in the distal esophagus and removed.

**Conclusions:**

Failure to consider body stuffing and foreign body impaction in individuals during medical evaluation in detention centers with complaints of chest pain, abdominal pain, dysphagia, and/or certain toxidromes can delay diagnosis and lead to a variety of medical complications.

## Introduction

Body stuffing is defined as oral ingestion of illicit drugs to avoid detection when individuals are about to be apprehended by law enforcement [1]. Small drug-containing packets can also be placed in the rectum or vagina [1, 2]. The drugs are usually poorly wrapped, so overdose still remains as a major concern. On the other hand, body packing is the planned and relatively well-coordinated ingestion of large quantities of drug for the purposes of smuggling [3, 4]. Therefore, body packers are at a higher risk of severe toxicity than body stuffers since they ingest much higher amounts of packets containing drugs [3, 4]. The method of packaging of each particular drug is very important, as there are differences in rates of complications depending on the packaging methods. Low-quality packages (condoms, toy balloons) filled with loose powder and tied with weak knots are rare (prevalence 9%), but are associated with much higher medical complications (prevalence 62.5%) [5]. Body stuffers are often not planning to ingest the illicit substance, and the packaging is more likely to be hurried, which may be more likely to lead to medical complications [6].

Most cases of body packers and stuffers who have swallowed drug packets are asymptomatic and do not get discovered by law enforcement personnel and as a result do not receive any medical attention. Usually, unruptured packets in the gastrointestinal tract are passed in stools [3]. There have been limited case reports on body packers or stuffers. Most cases are concerned about gastrointestinal complications such as colonic perforation [3]. In cases of suspected body packers and stuffers, law enforcement officials usually transport the individuals to the designated medical centers for further evaluation [3].

Previous case reports have focused on foreign body ingestion distal to the esophagus, and there are very few cases discussing complications of long-term esophageal impaction [7]. These complications may include erosion, tissue necrosis, and perforation. We present a case of an atypical presentation of a young adult with persistent epigastric abdominal pain due to an impacted esophageal packet of illicit drug. This case describes an intentionally swallowed plastic bag containing marijuana for concealment during apprehension by the law enforcement and adds to the scant literature regarding this subject.

## Case presentation

A 16-year-old Hispanic male was booked into a county juvenile detention and rehabilitation facility. Upon arrival at the facility and during the medical screening, he complained of persistent intractable epigastric pain, along with pleuritic chest pain with multiple episodes of vomiting over the previous 4 days. He further complained of dysphagia and odynophagia. At the time, he denied any past medical history and/or swallowing any foreign body. He admitted to smoking marijuana on a daily basis. During the initial care, he was given ondansetron (Zofran) 4 mg tablets and 30 mg of aluminum hydroxide/magnesium solution as well as two hydroxide/simethicone tablets orally.

**Fig. 1:**
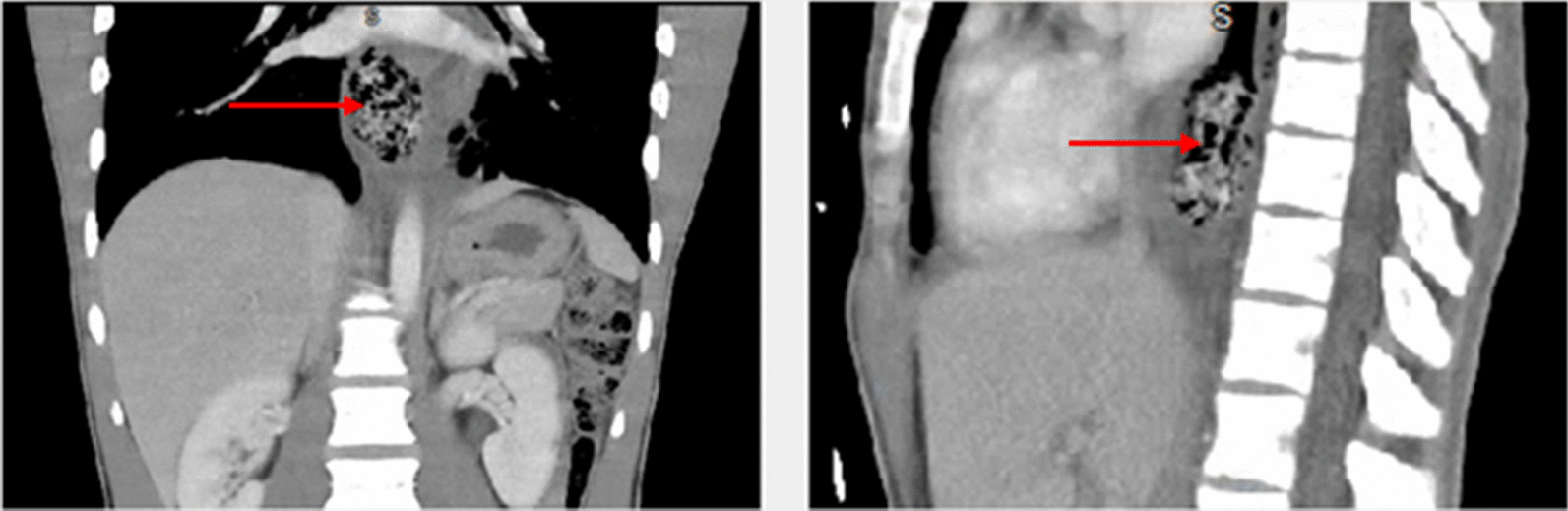
The red arrows on the computed tomography scans demonstrate distal esophageal foreign body with surrounding soft tissue edema

The patient subsequently became more subjectively comfortable, but the chest pain continued to persist. An electrocardiogram (EKG) was done during the same period and was evaluated by the on-call physician as being suspicious for acute pericarditis (Fig. 1).

**Fig. 2:**
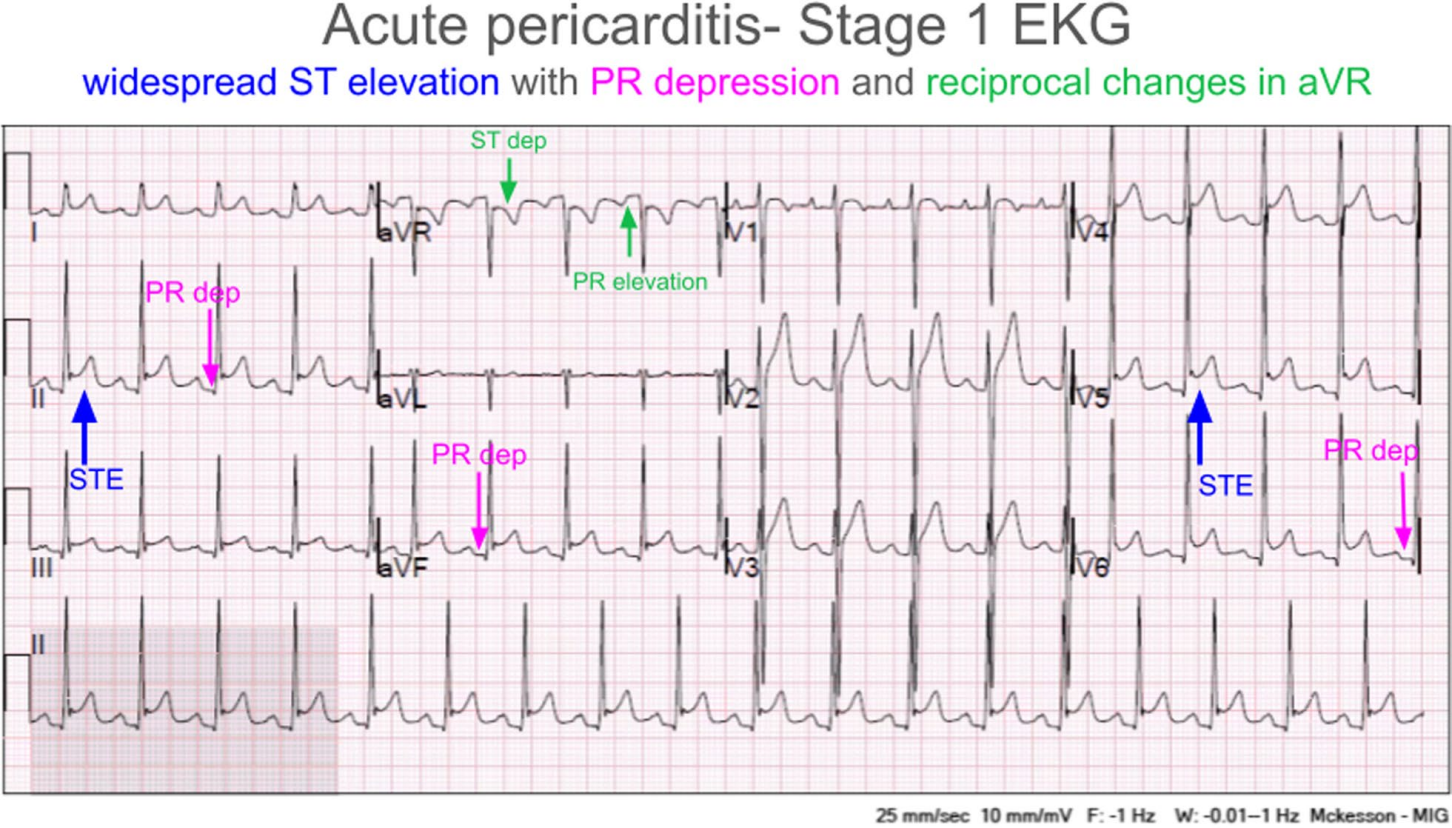
Twelve-lead EKG consistent with acute pericarditis. Red up arrow indicates diffuse concave ST elevations. Blue down arrow indicates hyperacute T waves. Plus symbol indicates PR depressions

On the basis of the assessment by the on-call physician, he was transferred to a local emergency department (ED) for further evaluation. In the first ED, physical examination indicated a normotensive, mildly tachycardic patient with moderate epigastric pain and otherwise normal physical examination. He admitted that his vomiting has improved but he still continues to have dysphagia. The patient underwent a series of laboratory tests that included complete blood count, serological liver function tests, a complete serum chemistry panel, and a computed tomography (CT) of abdomen and pelvis with intravenous contrast. The initial laboratory findings were significant for a mild leukocytosis, and the CT revealed pericholecystic fluid, distal esophageal edema, and dilation with questionable air in the esophageal wall, and possible hiatal hernia (Fig. 2).

The repeated EKG in the ED confirmed the possible diagnosis of pericarditis as well as concern for possible cholecystitis. Subsequently, the patient was transferred for a higher level of care to a regional medical center for further evaluation. During the course of care at the new ED, the patient continued to present with tachycardia, nausea, vomiting, and persistent epigastric and chest pain. The patient’s EKG continued to show diffuse ST elevation, while his cardiac markers remained within normal limits. Chest X-ray revealed a questionable infiltrate. Formal right upper quadrant ultrasound was performed and showed gallbladder wall thickening near the fundus of unclear etiology but no gallstones and was otherwise normal. His laboratory results were significant for white blood cells 12.5 × 10^9^ per liter, erythrocyte sedimentation rate 28 millimeters per hour, C-reactive protein 21.77 milligrams per decilitre, and procalcitonin 11.02 nanograms per milliliter, all elevated. Troponin and the remainder of the complete blood count and basic metabolic panel were grossly unremarkable.

The patient was treated with 2 liter of normal saline intravenously and 4 mg Zofran intravenously. Pain medication of hydrocodone/acetaminophen 5/325 mg orally as well as famotidine (Pepcid) 20 mg orally and gastrointestinal (GI) cocktail (aluminum hydroxide/magnesium hydroxide/lidocaine) 30 ml orally were given. Additionally, the patient was given ceftazidime 1 g intravenously and azithromycin 500 mg orally. While in the ED, he continued to deny swallowing a foreign body or ingesting chemicals that may have caused his symptoms. Upon further review of the patient’s symptoms, imaging studies, laboratory results, and recommendation by the surgery team, the patient was transferred to a nearby pediatric subspecialty hospital for further evaluation by pediatric surgery and gastroenterology and possible endoscopic evaluation of his esophagus.

**Fig. 3:**
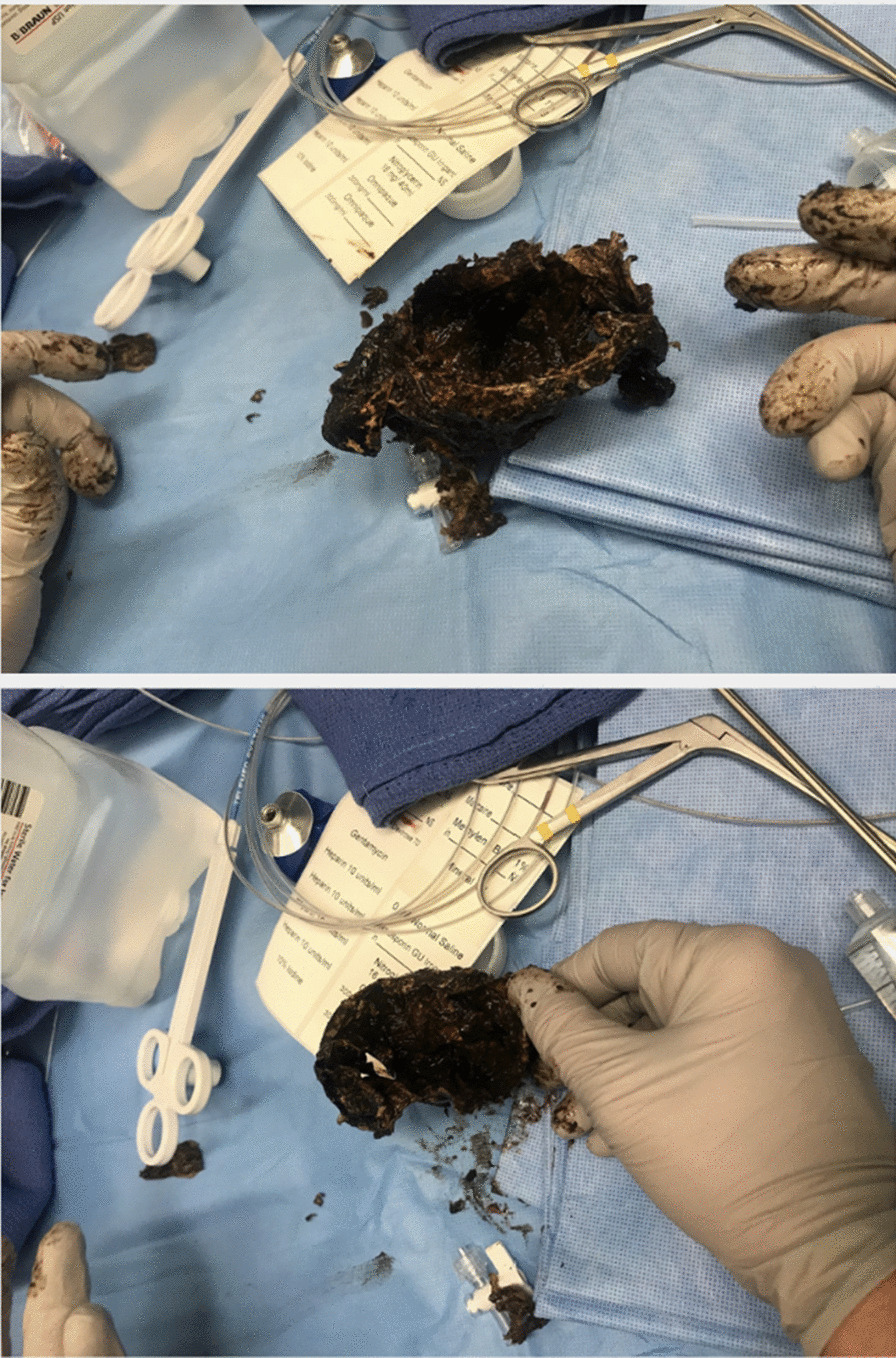
Bagged marijuana removed via endoscopy

On arrival to the pediatric subspecialty hospital, he continued to be tachycardic, but afebrile. In the ED, he received 4.5 gram (g) piperacillin/tazobactam (Zosyn), 20 mg Pepcid, and 4 mg Zofran, all intravenously. He immediately underwent an esophagram with water-soluble contrast that revealed a filling defect in the distal esophagus. After further questioning by the pediatric surgery team, he admitted that he had swallowed “a bag” prior to his arrest but would not disclose the contents of the bag. Subsequently, he underwent esophagogastroduodenoscopy, and a plastic bag with content suspicious for marijuana was discovered in the distal esophagus and removed (Fig. 3). Further analysis confirmed the presence of marijuana in the retrieved bag.

Patient was hospitalized with antibiotic therapy with intravenous Zosyn 3.375 g every 8 hours for suspected aspiration pneumonia and transitioned to oral amoxicillin/clavulanate (Augmentin) 875 mg tablets on the third day post-procedure for an additional 7 days of treatment. The patient also continued on 20 mg oral Pepcid twice a day. Further evaluation by pediatric cardiology and repeated EKG confirmed the diagnosis of pericarditis, likely post-infectious in etiology. His esophagus also showed evidence of achalasia, which was attributed to the prolonged irritation of the epithelial surface with the foreign body and the inflammation in the surrounding region. The patient was treated with ibuprofen (Motrin) 600 mg orally every 8 hours for the pericarditis. Furthermore, cardiology recommended outpatient follow-up in 2 months along with referral to pediatric rheumatology if symptoms persisted to explore other potential causes. The patient was discharged 3 days after the hospitalization and planned for a follow-up with outpatient pediatric surgery service 1 week later.

While awaiting for the outpatient follow-up, 7 days after discharge, he returned to the local ED with persistent left-sided chest pain and dyspnea worsened with exertions and wheezing. The patient was noted to have significant leukocytosis, and a CT of his chest revealed a left-sided loculated hydropneumothorax with central cavitary lesion. He was then transferred back to the pediatric surgery service at the same subspecialty hospital for further evaluation. Given concern for esophageal perforation, a repeat esophagram was performed showing normal esophageal caliber and contour without extravasation of contrast. The patient was then admitted to pediatric service with placement of a thoracostomy tube by the interventional radiology service. He received 4 days of thrombolytic therapy via the thoracostomy with satisfactory output. The thoracostomy tube was removed 10 days later. Infectious disease was also consulted and agreed with an antibiotic regimen of intravenous vancomycin, ceftriaxone, and clindamycin during his course in the hospital. The patient experienced resolution of his pericarditis symptoms with normalization of the EKG after the removal of the thoracostomy tube and was discharged home on continuation of intravenous ceftriaxone and vancomycin via peripherally inserted central catheter line as well as oral clindamycin.

## Discussions and conclusion

Esophageal foreign bodies are common in children and specific groups of adults, such as prisoners, developmental delay, psychiatric illnesses, and those dependent on alcohol [8]. Most of these patients are not forthcoming with information, so foreign body should be considered when high-risk individuals present with dysphagia, chest pain, or epigastric pain. Delayed or missed diagnosis of a foreign body ingestion can have significant morbidity, including airway obstruction and esophageal perforation [9]. Symptoms may not be present for an extended period of time even in the case of esophageal impaction [9].

Another potential consequence of missed body packing or body stuffing is toxic levels or even overdose of the ingested substance. Although cannabis is overall considered a relatively benign drug, there have been cases of neurologic complications and even death from cannabis body packing [7, 10].

The most common sites of obstruction in the esophagus are the cricopharyngeus muscle (75%), followed by the aortic arch and the lower esophageal sphincter [9]. The main risk factors for developing complications after foreign body ingestion were esophageal impaction that persists more than 24 hours, a positive radiographic finding, and patient age greater than 50 years [11]. Once the foreign body has made it past the stomach, the most common impaction point is the ileocecal valve, followed by the hepatic and splenic flexures [12]. If the object enters the stomach, expectant management is appropriate unless the object exceeds certain dimensions. A diameter greater than 2 cm will have trouble passing the pylorus and ileocecal valve. A length greater than 5 cm will have trouble making it through the curves of the duodenum [6].

Certain foreign bodies deserve special consideration, including button batteries; these can perforate the esophagus within 4 hours and need to be removed as soon as possible. Once the button battery is in the stomach, expectant management can be utilized; however, if it remains for longer than 36–48 hours or begins to cause symptoms, it must also be removed endoscopically [6]. Another category that deserves special consideration is sharp esophageal foreign bodies. These foreign bodies need to be removed endoscopically as there is a higher risk of perforation and the esophagus can then be evaluated for damage while the camera is there [6]

Workup is relatively simple; often, plain films are sufficient. The preferred screening tool is X-ray of the targeted area, with reported sensitivity of 47–95% for the initial radiographic evaluation and detection of the packed or stuffed illicit drugs or foreign bodies [13]. It is important to note that radiopaque objects can be missed on X-ray. CT scanning, ultrasound, and magnetic resonance imaging can be utilized to detect the foreign body; however, false-negative abdominal CT scans have been reported in previous publications, based on the materials used to make the packages containing the drugs [12].

However, this is assuming that healthcare providers are aware of a foreign body ingestion in the first place. Failure to make this diagnosis can lead to long-term complications, including esophageal rupture, perforation, and aspiration pneumonia [6]. Timely diagnosis is imperative as all foreign bodies in the esophagus should be removed via endoscopy within 24 hours to prevent these complications [11]. Ascertaining the type of drug ingested and the material used to wrap the packages will also play a role in how urgently an endoscopy must be performed [5]. This case demonstrates many of the complications that can occur from long-term esophageal impaction.

Management of foreign bodies in the ED includes expectant management, medications, and attempts at mechanical removal. If these attempts fail or are not viable options, the next steps are endoscopy and surgery [14]. Intravenous glucagon (1–2 mg, can be repeated once) has differing success rates (12–50%) and tends to work better with distal foreign bodies as it relaxes muscle [14]. Use of benzodiazepines may assist secondary to striated muscle relaxation. Many other medications have not been shown to help, including calcium channel blockers, nitrates, and anticholinergic medications [14]. Foley catheter can be successful within 72 hours; this requires sedation and fluoroscopy while being ready for intubation if necessary [15]. Endoscopy is the gold standard for esophageal and gastric foreign body retrieval and should be the modality of choice if available. When unsuccessful, the foreign object is usually small and sharp, and surgery may be required in such cases [14, 16].

Ingested foreign bodies can be difficult to diagnose and treat in detention centers and ED settings, especially in the context of patient populations that may not be cooperative or forthcoming with the information. It is critical to maintain a high index of suspicion and readiness to intervene for this potentially life-threatening presentation. This case reveals many challenges involved in the management of the uncooperative patients with swallowed bags of illicit drugs. It is important to keep foreign bodies on the differential for high-risk patients as a cause of atypical chest pain, persistent nausea, dysphagia, and epigastric pain.

## Data Availability

Not applicable.

## Abbreviations

EKG: Electrocardiogram
ED: Emergency department
CT: Computed tomography
mg: milligram
g: gram
