# Correction: Body stuffing during apprehension resulting in distal esophageal impaction: a case report and review of the literature

**DOI:** 10.1186/s13256-023-03938-6

**Published:** 2023-04-20

**Authors:** Tegan Schmidt, Yuliya Matolina, Arianna S. Neeki, Carlos Peace, Benjamin Archambeau, Fanglong Dong, Michael M. Neeki

**Affiliations:** 1 Department of Emergency Medicine, Arrowhead Regional Medical Center, 400 N. Pepper Ave, Colton, CA 92324 USA; 2 Department of Probations, San Bernardino County, San Bernardino, CA 92415 USA; 3 California Universityof Science and Medicine, Colton, CA 92324 USA

**Correction** **:** **Journal of Medical Case Reports (2022) 16: 426** **https://doi.org/10.1186/s13256-022-03628-9**

Following publication of the original article [1], the authors identified an error in the author name of Carlos Peace.

The incorrect author name is: Caros Peace.

The correct author name is: Carlos Peace.

The author group has been updated above and the original article [1] has been corrected.

## References

[1] Schmidt T, Matolina Y, Neeki AS, Peace C, Archambeau B, Dong F, Neeki MM (2022). Body stuffing during apprehension resulting in distal esophageal impaction: a case report and review of the literature. J Med Case Rep.

